# Summer Schools

**Published:** 1860-06

**Authors:** Henry F. Andrews

**Affiliations:** Late House Physician to Bellevue Hospital.; Washington, Ga.


**Summer Schools.**

WASHINGTON, GA., April 20th, 1860.

*Editors of the New York Medical Press:*

In No. 13 of the *Press* I see an article in regard to the L. I. College Hospital, in which it is stated that some of the older schools of your city refuse to recognise it or any other Summer School.

Like yourselves, gentlemen, I am unable to discover the reason. I cannot see what difference it makes where a student attends his first course, provided it be not in a homœpathic or other quack institution, if he is able to pass a creditable examination when he applies for his degree.

I have always thought it wrong for our colleges to make three years study and two full courses of lectures the chief requisites for graduation. Why do they attach more importance to these than to more substantial qualifications? How much better would it be to make candidates for graduation stand upon merit and merit alone.

When I came before the President of the Faculty to stand my examination on his branch, he gave me a paper to sign, certifying that I was twenty-one years of age, had read medicine three years under a regular practitioner, and had attended two full courses of lectures. I know many who received their diplomas at the same time, whose principal qualification was their signature to the certificate, and who, for the good of humanity and the honor of the profession, ought this day to be working at some useful trade, or engaged in some occupation in which little intellect or education is necessary. And yet the college ranks high, is as strict as the generality of such institutions, and is, altogether, an excellent school for acquiring a medical education.

I sometimes think it is cupidity which causes our colleges to require two courses, and to graduate so many undeserving candidates. There also seems to be, combined with this cupidity, an ambition to graduate as large a number as possible; indeed, there seems to be a rivalry between some to see which can turn out the largest number of doctors annually. How much more laudable an ambition would it be to strive to graduate the best, most accomplished and skilful physicians!

Is not a man of fine talent, who has received a good literary education, who has studied his medical text-books thoroughly for twelve months, or even less, who has attended one course of lectures, and who is able and ready to stand a strict and thorough examination, infinitely better prepared to practice our noble profession than the dunce with little or no education, who stumbles through the farce of an examination, but who can write his name upon the all-important piece of foolscap!

Yet how many of the latter class have I seen receive their diplomas and go forth from their *Alma Mater* upon the same footing with, and without distinction from, the former, a disgrace to our noble calling, and a stigma upon the institution which graduated them.

Is it any wonder that the public is daily becoming more and more skeptical in regard to the efficacy of medical science? Is it any wonder that our profession, our diplomas, nay, our very titles, are continually the subjects of low, coarse and vulgar jests? Is it any wonder that vaunting quacks of every description are spread, like a continued pestilence, over the whole face of our fair land? Truly hath it been said—

> "Death dealing doctors devastate the land,
> And spread disease with unrelenting hand."

I notice in No. 12 of the *Press* an article on medical advertising by my former class-mate and hospital colleague, Dr. Charles Haase. I do not disapprove his plan, but I think the most effectual remedy against quackery is first to create reform and great reform amongst ourselves. Let every graduate who leaves his college halls go fully prepared to shoulder the mighty responsibilities of a medical practitioner; let every one be fully skilled and versed in every branch of his profession, and quackery, in a few years, will be an almost obsolete word.

Do the professors of our colleges ever reflect upon the criminality of graduating so many unworthy candidates? Do they ever reflect upon the number of murders they are indirectly guilty of?

Another great objection to the present system is,—there are many worthy and talented young men who are pecuniarily unable to attend more than one course of lectures, but who might qualify themselves fully by one. Let such a one however, attempt to practice, and he is considered a quack; the diplomatised will refuse to meet him in consultation, and will never, by word or deed, acknowledge him to be a brother, or admit him to any of the privileges of a physician. Yet his next door neighbor, though a dunce, is admitted into the ranks, and has the right hand of fellewship extended to him, why? He possessed the *sine qua non*, the talisman which alone could carry him through the terrors of the green room; he could sign a certificate, that he was twenty-one years ot age, had studied medicine three years under a regular practitioner, and had attended two full courses of lectures!

Oh! most worthy, grand, and dignified Professor, who didst so often, in words of glowing eloquence, impress upon our youthful minds the terrible resposibilities of our profession and the requirements necessary to its pursuit! What a profound, philosophical, and sensible test you made use of to discover whether we were properly prepared to assume these responsibilities, whether we possessed those necessary acquirements!

Now, Messrs. Editors, can you inform me what is the difference where a candidate for graduation has attended his first course, or whether he has attended more than one course or not, provided he be worthy a diploma?

The University of Virginia is, undoubtedly, conducted on the best plan. There, a student graduates at the close of his first session, provided he pass his examination. But there are very few, out of a large number of candidates, who receive diplomas; those few, however, are most thoroughly accomplished physicians, so far as education is concerned, the very day they obtain their degree. And how much better, more pleasant and creditable it would be to have our profession composed of such men than crowded and overstocked with stupid, uneducated blockheads, who, too often, are knaves as well as dunces.

Excuse me, gentlemen, for consuming so much of your valuable space, but I feel deeply interested in the subject, and though I cannot hope with my feeble pen to accomplish anything, yet it is a relief to utter my sentiments freely.

HENRY F. ANDREWS, M. D.,
Late House Physician to Bellevue Hospital.

